# Oatmeal, Bone and Muscle

**Published:** 1874-02

**Authors:** 


### OATMEAL, BONE AND MUSCLE.

Liebig has shown that oatmeal is almost as nutritious as the very best English beef, and that it is richer than wheaten bread in the elements that go to form bone and muscle. Professor Forbes, of Edinburgh, during some twenty years, measured the breadth and height, and also tested the strength of both the arms and loins of the students in the University—a very numerous class, and of various nationalities, drawn to Edinburgh by the fame of his teaching. He found that, in height, breadth of chest and shoulders, and strength of arms and loins, the Belgians were at the bottom of the list ; a little above them the

French ; very much higher, the English ; the highest of all, the Scotch and Scotch-Irish, from Ulster, who, like the natives of Scotland, are fed, in their early years, with at least one meal a day of good milk and good oatmeal porridge. A very good drink is made by putting about two spoonfuls of the meal into a tumbler of water. The Western hunters and trappers consider it the best of drinks, as it is at once nourishing, unstimulating and satisfying.—*Med. Investigator.*

